# Risk Prediction of Major Adverse Cardiovascular Events Occurrence Within 6 Months After Coronary Revascularization: Machine Learning Study

**DOI:** 10.2196/33395

**Published:** 2022-04-20

**Authors:** Jinwan Wang, Shuai Wang, Mark Xuefang Zhu, Tao Yang, Qingfeng Yin, Ya Hou

**Affiliations:** 1 School of Information Management Nanjing University Nanjing China; 2 First Department of Cardiology The Affiliated Hospital of Liaoning University of Traditional Chinese Medicine Shenyang China; 3 School of Artificial Intelligence and Information Technology Nanjing University of Chinese Medicine Nanjing China; 4 Jiangsu Famous Medical Technology Co Ltd Nanjing China

**Keywords:** major adverse cardiovascular events, risk prediction, machine learning, oversampling, data imbalance

## Abstract

**Background:**

As a major health hazard, the incidence of coronary heart disease has been increasing year by year. Although coronary revascularization, mainly percutaneous coronary intervention, has played an important role in the treatment of coronary heart disease, major adverse cardiovascular events (MACE) such as recurrent or persistent angina pectoris after coronary revascularization remain a very difficult problem in clinical practice.

**Objective:**

Given the high probability of MACE after coronary revascularization, the aim of this study was to develop and validate a predictive model for MACE occurrence within 6 months based on machine learning algorithms.

**Methods:**

A retrospective study was performed including 1004 patients who had undergone coronary revascularization at The People’s Hospital of Liaoning Province and Affiliated Hospital of Liaoning University of Traditional Chinese Medicine from June 2019 to December 2020. According to the characteristics of available data, an oversampling strategy was adopted for initial preprocessing. We then employed six machine learning algorithms, including decision tree, random forest, logistic regression, naïve Bayes, support vector machine, and extreme gradient boosting (XGBoost), to develop prediction models for MACE depending on clinical information and 6-month follow-up information. Among all samples, 70% were randomly selected for training and the remaining 30% were used for model validation. Model performance was assessed based on accuracy, precision, recall, F1-score, confusion matrix, area under the receiver operating characteristic (ROC) curve (AUC), and visualization of the ROC curve.

**Results:**

Univariate analysis showed that 21 patient characteristic variables were statistically significant (*P*<.05) between the groups without and with MACE. Coupled with these significant factors, among the six machine learning algorithms, XGBoost stood out with an accuracy of 0.7788, precision of 0.8058, recall of 0.7345, F1-score of 0.7685, and AUC of 0.8599. Further exploration of the models to identify factors affecting the occurrence of MACE revealed that use of anticoagulant drugs and course of the disease consistently ranked in the top two predictive factors in three developed models.

**Conclusions:**

The machine learning risk models constructed in this study can achieve acceptable performance of MACE prediction, with XGBoost performing the best, providing a valuable reference for pointed intervention and clinical decision-making in MACE prevention.

## Introduction

The treatment of coronary heart disease has experienced major advances with respect to thrombolysis [1], percutaneous coronary intervention (PCI) [2], coronary artery bypass grafting (CABG) [3], and other modalities, which have significantly reduced the disability and mortality rate of coronary heart disease with increased efficacy and safety. Despite the mature use of coronary revascularization, the possible concomitant postoperative complications, including stent restenosis, stent thrombosis, coronary microvascular dysfunction, myocardium ischemic/reperfusion injury, depression/anxiety before and after surgery, and procedure-related vascular complications, have led to a high rate of major adverse cardiovascular events (MACE), with an incidence of approximately 15% to 25%, mainly occurring within 6 months or 12-18 months after the operation [4,5]. The occurrence of MACE is a serious issue that markedly affects the prognosis of patients; thus, developing methods to reduce or even avoid MACE has been a long-standing and imperative clinical challenge. Faced with these needs, a reliable risk prediction model of MACE after coronary revascularization can effectively predict the severity of disease to help clinicians and patients in the shared decision-making process of treatment and rehabilitation plans, which is of practical significance to take early measures so that interventions can be delivered early to reduce the probability of adverse events.

In recent years, there has been an explosion of studies on MACE risk assessment, which can be divided into rule-based expert systems, statistical-based analysis techniques, and machine learning (ML)-based prediction models [6]. As a representative expert system, the assistive diagnostic system MYCIN, developed by Shortliffe et al [7], uses predicate logic and first-order logic to imitate the reasoning process of an expert to identify bacterial infections and provide available treatment options. However, this medical expert system requires a manual summarization of a large number of expert rules, which leads to high maintenance costs and poor expansibility. In response to these problems, statistical analysis has been incorporated into medical data processing to aid clinical decision-making by exploring the relationship between target and explanatory variables [8,9]. With the continuous development of data mining, ML algorithms [10] have been gradually applied in the field of clinical medical research [11,12] by virtue of the powerful data processing and knowledge representation capabilities, achieving better predictive performance by deeply mining the inherent laws of data to obtain insight into the tendency of future development. Disease identification and prediction are often regarded by ML as a classification problem with clinical manifestations as feature variables and the corresponding diagnostic results as targeted labels. For example, Zhu et al [13] constructed a model for predicting the risk of central lymph node metastasis utilizing available preoperative characteristics and intraoperative frozen section information. Patel et al [14] developed a fast and efficient detection technique for heart disease based on 303 records with 76 attributes. Duan et al [15] proposed a novel approach of MACE prediction for patients with acute coronary syndrome using not only static patient features but also dynamic treatment information during their hospitalization, which appeared to boost the performance and readily meet the clinical prediction demand. 

These studies have indicated that ML has better predictive performance over conventional statistical approaches. Hence, it might be a better choice to develop a predictive model by capitalizing on the strong generalization and robustness of ML methods. However, in clinical reality, a nonnegligible problem is that the distribution of data is often imbalanced and can even be severely imbalanced in some cases [16]; that is, the number of samples with MACE occurrence is significantly smaller than that without MACE occurrence. In such a case, poor risk models may be obtained because the decision boundary is likely biased in response to the unbalanced data [17].

Data imbalance is a common clinical occurrence [18]. For example, in early cancer screening, the general population is much larger than the population of cancer patients [19]. In the identification of frailty in the elderly, the number of subjects from the negative sample far exceeds that of the positive sample [20]. Similarly, in considering risk prediction of MACE occurrence after coronary revascularization, there are relatively fewer patients with MACE occurrence than without. The challenge with using imbalanced data sets is that most ML techniques, which aim for overall classification accuracy, will ignore the minority class in model training, making the minority perform poorly [21]. In such a case, although high overall accuracy can be achieved, the recognition rate of the minority class is extremely low, which is usually more important. Sample reconstruction is a commonly used intervention for imbalanced classification to balance the positive and negative classes, mainly including undersampling and oversampling [22]. Undersampling aims to balance uneven data sets by removing data from the majority class and keeping all of the data in the minority class. Although it is a common and important approach, undersampling can somewhat affect the model performance as some potentially important information can be lost. Conversely, oversampling extends the size of the minority class by duplicating or synthesizing. This approach is appropriate when the original sample set does not contain sufficient information. The most frequently employed oversampling approach is the synthetic minority oversampling technique (SMOTE) [23], which has been successfully applied in imbalanced learning in various fields [24,25], including clinical research [26,27]. For example, Ishaq et al [27] proved that their model achieved the best performance on a data set that was balanced with the SMOTE technique in the prediction of survival for patients with heart disease. In addition to direct employment of the original SMOTE technique, some improved versions have been developed to synthesize higher-quality samples [28,29]. For example, Prusty et al [28] proposed the weighted-SMOTE approach, in which oversampling of each minority data sample is carried out based on an assigned weight.

Accordingly, to achieve risk prediction of MACE after coronary revascularization, the aim of this study was to establish prediction models using ML algorithms based on sufficient data processing. First, the SMOTE technique was adopted to balance the initial imbalanced data set. For model construction, six algorithms were respectively employed to build six predictive models, and then the optimal model was determined according to systematic comparison and evaluation. The models were then further explored to identify factors affecting the occurrence of MACE. This study can therefore provide a valuable reference for pointed intervention and clinical decision-making in MACE prevention.

## Methods

### Study Participants

We retrospectively collected the medical records of patients who underwent coronary revascularization at The People’s Hospital of Liaoning Province and Affiliated Hospital of Liaoning University of Traditional Chinese Medicine from June 2019 to December 2020, including clinical information and follow-up information within 6 months of surgery.

### Inclusion and Exclusion Criteria

The general inclusion criteria were as follows: (1) age ≥18 years and ≤85 years; (2) patients with previous coronary revascularization (including CABG and/or PCI).

Exclusion criteria were as follows: (1) patients with incomplete medical records and unable to provide original surgical information; (2) patients who had not undergone coronary revascularization or for whom the surgery failed; (3) patients who required mechanical assistive therapy with an intraaortic balloon pump (IABP) after successful coronary revascularization treatment, since these patients are critically ill, requiring IABP treatment to maintain vital signs and do not have indications for discharge or follow-up; (4) combined with other heart diseases such as malignant arrhythmia, cardiac insufficiency before and after surgery (ie, patients with New York Heart Association class IV or Killip class IV), or severe cardiopulmonary insufficiency, as these patients are in a severe condition and have underlying diseases resulting in a poor prognosis or even surgical intervention, leading to lack of follow-up or are already at the endpoint before enrollment; and (5) patients with neuropathy or those who may not be able to participate in the study due to literacy, language, or other communication barriers.

### Data Exploration

Before data modeling, data analysis and preprocessing are indispensable [30]. We used the occurrence of MACE within 6 months after coronary revascularization as the study endpoint. For this study, MACE was defined to involve all-cause deaths, nonfatal myocardial infarction, recurrent angina, repeat revascularization, stroke, and readmission within 6 months after coronary revascularization. The total number of characteristic variables in the raw data set was 49, which mainly involved five aspects: subject characteristics, medical history, drug prescriptions, clinical events, and clinical psychiatric evaluations. Initially, removing the records with null clinical endpoints and those with more than 80% missing features, we obtained a data set containing 1004 records, including 753 without MACE and 251 with MACE. Subsequently, eight unimportant characteristic variables with high missing rates (over 60%) were deleted through communication with clinical experts, and the missing values of the remaining 41 characteristic variables, if any, were filled in. The specific data-filling approach was as follows. First, we logged into the Data Management Center of Jiangsu Famous Medical Technology Co Ltd, the cloud storage platform for the data, to search for missing values, because there may have been a system failure during the data export process. If not available, we would continue to search for paper copies of the original information and records. If these were not found, data-filling methods were employed [31] using the expectation-maximization algorithms for continuous variables and the mode for filling in missing data of discrete variables.

The preliminary exploratory analysis revealed that the original data set had a category imbalance problem; that is, the ratio of the number of samples with and without MACE was approximately 1:3, which would affect the performance of the final risk prediction model to a certain extent. Therefore, we determined that the original data set should first be processed by equalization. Currently, the main strategies to solve the imbalanced classification problem include oversampling and undersampling [32], among which the former, represented by SMOTE [23,33], is widely believed to be an effective strategy for resolving class imbalance. Therefore, we adopted SMOTE for sample reconstruction in this study.

### SMOTE Technique

SMOTE is a novel oversampling technique proposed by Chawla et al [23], which has become an effective preprocessing technique for uneven data sets. In contrast to many traditional oversampling methods, SMOTE does not simply duplicate the samples but rather increases the number in the minority class by creating new synthetic samples. This reduces the likelihood of overfitting and improves the generalization performance of the classifier on the test set. The algorithm flow is as follows [23]:

(1) For each sample *x* in the minority class, calculate the Euclidean distance between *x* and all samples in the minority class and obtain its k-nearest neighbors.

(2) Select several samples from the k-nearest neighbors of *x* at random.

(3) For each randomly selected neighbor *x_n_*, a new sample is synthesized according to the formula:

x_new_= x+rand(0,1)×(x_n_–x)

Depending on the sampling rate, we set the execution time and repeated the above process. Finally, we obtained the final minority class by combining the synthetic samples with the original samples.

### Research Technique

All data were statistically analyzed with SPSS 26.0 software. The enumeration data are expressed as count (percentage), processed with a *χ*^2^ test, whereas the measurement data are presented as means (SD) and analyzed by *t* tests. A *P* value less than .05 was accepted to indicate statistical significance.

ML algorithms are characterized by better performance compared with traditional statistical methods in risk prediction, which were selected for modeling in MACE prediction in this study. We randomly separated the entire data set into a training set and validation set with an approximate ratio of 7:3, in which the training set was used to construct the prediction model and the validation set was used to verify and evaluate the model performance. Six ML algorithms were employed to construct risk prediction models: decision tree (DT), random forest (RF), logistic regression (LR), naïve Bayes (NB), support vector machine (SVM), and extreme gradient boosting (XGBoost). Among them, RF and XGBoost are ensemble ML classifiers and the others are single classifiers. Throughout the experiment, we implemented modeling and evaluation using Python 3.8 with open-source Python libraries. During the training process, the optimal parameters were determined by 10-fold cross-validation to prevent overfitting, and then we obtained the final ML-based risk models of MACE prediction.

### Evaluation Metrics

The performance of ML models is often assessed with certain evaluation metrics [34]. The blend of various evaluation metrics is expected to facilitate analytical research [35]. In this study, the indicators accuracy, precision, recall, F1-score, and area under the receiver operating characteristic (ROC) curve (AUC) were all employed for model evaluation. Values closer to 1 for these metrics indicate better performance of the predictive models. We also used the ROC curve as a common measure to graphically visualize the discriminative power of models.

For classification tasks, the confusion matrix [34] is also a critical index in model evaluation. The confusion matrix for binary classification is shown in Table 1.

Based on the confusion matrix, the values of the other evaluation metrics can be readily calculated, as follows:

Accuracy=(TP+TN)/(TP+FP+TN+FN)

Precision=TP/(TP+FP)

Recall=TP/(TP+FN)

F1-score=2×TP/(2×TP+FP+FN)=2×precision×recall​/(precision+recall)

Where TP is true positive, TN is true negative, FP is false positive, and FN is false negative.

**Table 1:** The confusion matrix for binary classification.

Labeled	Predicted as negative	Predicted as positive
Negative	True negative	False positive
Positive	False negative	True positive

### Ethics Approval

This study was approved by the Institutional Review Board of The Affiliated Hospital of Liaoning University of Traditional Chinese Medicine (2019034FS(KT)-016-02).

## Results

### Univariate Analysis

Based on expert experience, a total of 1004 samples with 41 characteristic variables were finally adopted for model construction after data preprocessing, including 251 cases with MACE and 753 cases without MACE. The detailed statistical information of the feature variables and results of the univariate analysis of MACE are shown in Table 2. Due to space limitations, only the statistically significant characteristic variables are presented, and the complete information of the 41 variables is provided in Multimedia Appendix 1. The results revealed that 21 characteristics were significantly different (*P*<.05) between the groups without and with MACE, namely age, smoking, work, course of the disease, family history, seasonal onset, previous myocardial infarction, dyslipidemia, brain infarction, cardiac insufficiency, traditional Chinese medicine (TCM) treatment, anticoagulant drugs, antiarrhythmic drugs, diuretic, lansoprazole injection, bleeding events, left atrial diameter (LAD), left ventricular ejection fraction (LVEF), bypass surgery, Hamilton anxiety scale (HAMA), and Hamilton depression scale (HAMD).

**Table 2:** Significant variables in univariate analysis.

Characteristics		Without MACE^a^ (n=753)	With MACE (n=251)	Statistic^b^		*df*		*P* value	
Age (years), mean (SD)		63.47 (10.98)	66.82 (11.10)	–4.180		1002		<.001	
**Smoking, n (%)**					4.360		1		.04
	No	429 (57.0)	124 (49.4)						
	Yes	324 (43.0)	127 (50.6)						
**Type of work, n (%)**					6.213		1		.013
	Physical work	353 (46.9)	95 (37.8)						
	Mental work	400 (53.1)	156 (62.2)						
Course of disease (years since diagnosis), mean (SD)		3.41 (5.11)	5.43 (5.81)	–4.930		387.17		<.001	
**Family history, n (%)**					4.387		1		.04
	No	693 (92.0)	220 (87.6)						
	Yes	60 (8.0)	31 (12.4)						
**Seasonal onset, n (%)**					17.920		1		<.001
	No obvious seasonality	675 (89.6)	199 (79.3)						
	Obvious seasonality	78 (10.4)	52 (20.7)						
**Previous myocardial infarction, n (%)**					80.775		1		<.001
	No	643 (85.4)	147 (58.6)						
	Yes	110 (14.6)	104 (41.4)						
**Dyslipidemia, n (%)**					6.659		1		.01
	No	681 (90.4)	240 (95.6)						
	Yes	72 (9.6)	11 (4.4)						
**Brain infarction, n (%)**					24.822		1		<.001
	No	671 (89.1)	192 (76.5)						
	Yes	82 (10.9)	59 (23.5)						
**Cardiac insufficiency, n (%)**					6.249		1		.01
	No	712 (94.6)	226 (90.0)						
	Yes	41 (5.4)	25 (10.0)						
**TCM** ^c^ **treatment, n (%)**					4.489		1		.03
	No	570 (75.7)	173 (68.9)						
	Yes	183 (24.3)	78 (31.1)						
**Anticoagulant drugs, n (%)**					47.408		1		<.001
	No	367 (48.7)	185 (73.7)						
	Yes	386 (51.3)	66 (26.3)						
**Antiarrhythmic drugs, n (%)**					4.123		1		.04
	No	703 (93.4)	243 (96.8)						
	Yes	50 (6.6)	8 (3.2)						
**Diuretic, n (%)**					6.055		1		.01
	No	636 (84.5)	195 (77.7)						
	Yes	117 (15.5)	56 (22.3)						
**Lansoprazole injection, n (%)**					14.381		1		<.001
	No	634 (84.2)	235 (93.6)						
	Yes	119 (15.8)	16 (6.4)						
**Bleeding events, n (%)**					12.446		1		<.001
	No	735 (97.6)	233 (92.8)						
	Yes	18 (2.4)	18 (7.2)						
LAD^d^ (mm), mean (SD)		36.59 (4.91)	37.70 (5.54)	–2.988		1002		.003	
LVEF^e^ (%), mean (SD)		52.65 (8.08)	51.31 (8.91)	2.113		395.97		.04	
**Bypass surgery, n (%)**					7.200		1		.007
	No	745 (98.9)	242 (96.4)						
	Yes	8 (1.1)	9 (3.6)						
HAMD^f^, mean (SD)		7.23 (5.26)	9.27 (5.87)	–4.877		392.12		<.001	
HAMA^g^, mean (SD)		8.23 (6.59)	11.13 (6.83)	–5.979		1002		<.001	

^a^MACE: major adverse cardiovascular events.

^b^*t* statistics for continuous variable comparisons and *χ*^2^ statistics for categorical variables.

^c^TCM: traditional Chinese medicine.

^d^LAD: left atrial diameter.

^e^LVEF: left ventricular ejection fraction.

^f^HAMD: Hamilton depression scale.

^g^HAMA: Hamilton anxiety scale.

### Oversampling

To cope with data imbalances in the original data sets, the SMOTE algorithm was employed. The sample distribution before and after oversampling is shown in Table 3. The ratio of sample numbers with and without MACE occurrence was 1:1 after oversampling for both the training and validation set.

**Table 3:** Data distribution before and after oversampling.

Oversampling	Training set		Validation set	
	Without MACE^a^	With MACE	Without MACE	With MACE
Before	527	176	226	75
After	527	527	226	226

^a^MACE: major adverse cardiovascular events.

### Modeling and Evaluation

Taking whether MACE occurred within 6 months as the label and 21 statistically significant factors in the univariate analysis as features, the MACE risk prediction models were constructed by DT, RF, LR, NB, SVM, and XGBoost, respectively. As the central aspect, model evaluation is quite essential. First, we comprehensively compared ML algorithms before and after oversampling to test the effectiveness of the SMOTE strategy, with specific results presented Table 4. The performance of the ML models based on the oversampled data set was significantly better than that of models based on the original imbalanced dataset, thus demonstrating the rationality of the oversampling strategy. It is worth noting that although the accuracy before oversampling was slightly higher than that obtained after oversampling, other indicators such as precision, recall, F1-score, and AUC were significantly lower than those obtained after oversampling, especially precision, recall, and F1-score. The reason for the high accuracy before oversampling is that this comes at the expense of the accuracy of minority samples to improve the overall accuracy, which is of little significance for the imbalanced classification problem [36], whereas the evaluation indicators such as precision, recall, F1-score, and AUC should be more relevant than the overall accuracy on imbalanced issues.

**Figure 1:**
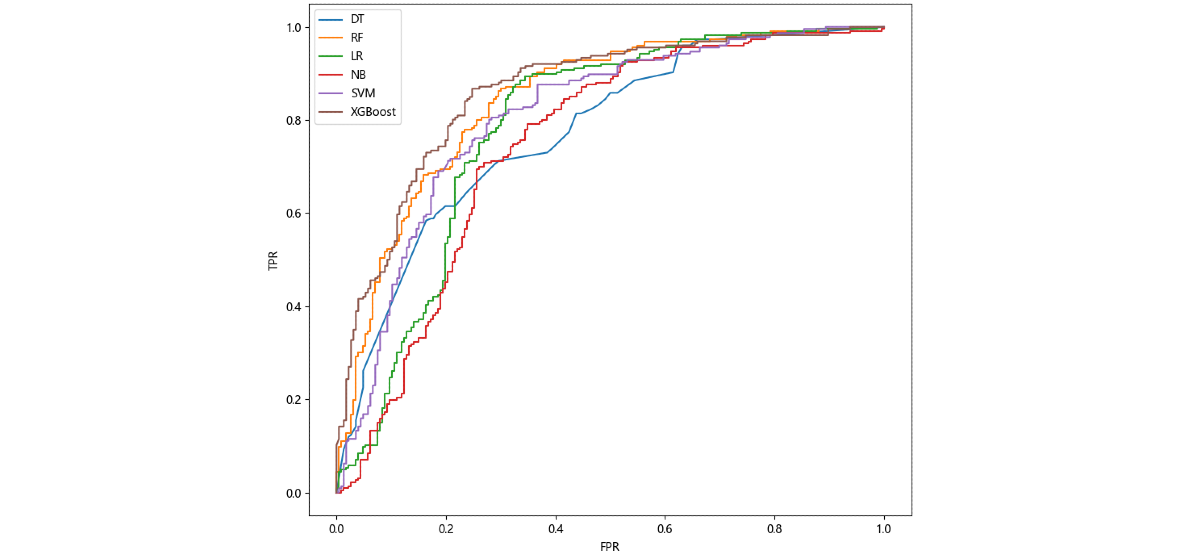
ROC curves of machine learning algorithms after oversampling. ROC: receiver operating characteristic; DT: decision tree; RF: random forest; LR: logistic regression; NB: naïve Bayes; SVM: support vector machine; XGBoost: extreme gradient boosting; TPR: true positive rate; FPR: false positive rate.

The ROC curves for the six ML algorithms based on balanced data sets are detailed in Figure 1. Combined with the results of Table 4, it is clear that XGBoost and RF had better performance with respect to accuracy, precision, F1-score, and AUC, with XGBoost having the best effect. This is likely because both XGBoost and RF belong to ensemble learning methods, with the advantages of integrating the performance of multiple weak classifiers. However, NB outperformed the other models in terms of recall. From the definition, as detailed above, recall is the proportion of correctly identified positive samples among all positive samples, which indicates that NB is more sensitive to positive samples than other models. However, as previously explained, no single indicator exists that can comprehensively evaluate a model’s performance. NB had the lowest values for all metrics except for recall. Therefore, it is clear that XGBoost achieved optimal performance in MACE prediction from an overall perspective, with an accuracy of 0.7788, precision of 0.8058, recall of 0.7345, F1-score of 0.7685, and AUC of 0.8599.

**Figure 2:**
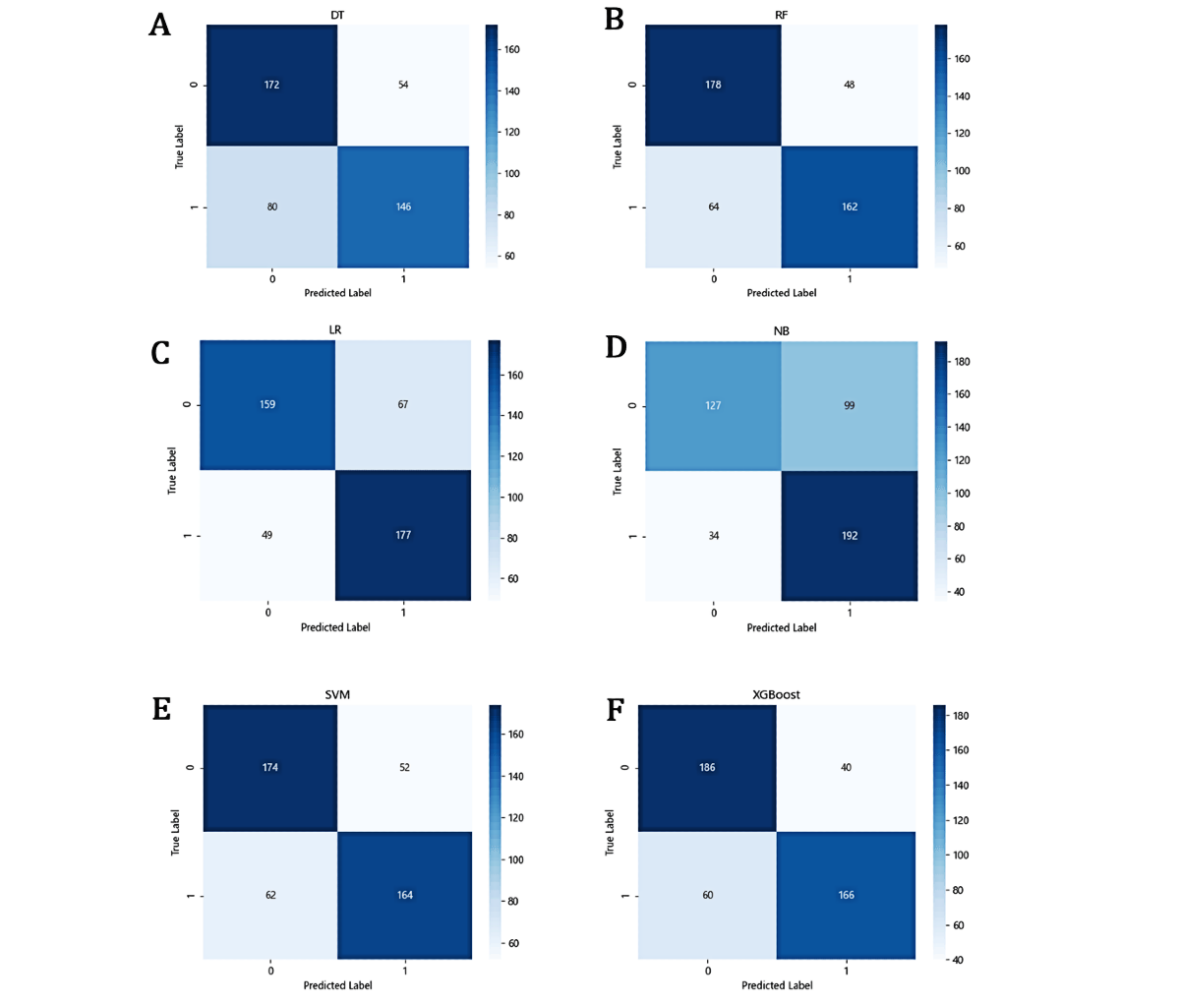
Confusion matrix of the risk prediction models with machine learning algorithms: (A) decision tree (DT), (B) random forest (RF), (C) logistic regression (LR), (D) naïve Bayes (NB), (E) support vector machine (SVM), (F) extreme gradient boosting (XGBoost).

As another method to assess the effectiveness of classification, the confusion matrices of all methods are illustrated in Figure 2. Specifically, 0 stands for the negative samples (ie, patients without MACE occurrence) and 1 represents the positive samples (ie, patients with MACE occurrence). It can be intuitively seen that DT, RF, SVM, and XGBoost had higher recognition rates for negative samples, with XGBoost performing the best. The fact that XGBoost had only 100 misclassified samples, which was the lowest among all models, further proving its superiority. In contrast, LR and NB had higher identification rates for positive samples, with NB accurately identifying 192 positive samples, confirming that NB is more sensitive to the minority class. However, NB only identified 127 negative samples, which was the lowest recognition rate of negative samples among all models. In addition, there were 133 misclassified samples with NB, which was second only to DT. Combining these results with those shown in Table 4, we can infer that NB is poor at identifying negative samples despite its high recall, which suggests that the classification boundary is biased toward the minority class (ie, patients with MACE).

**Figure 3:**
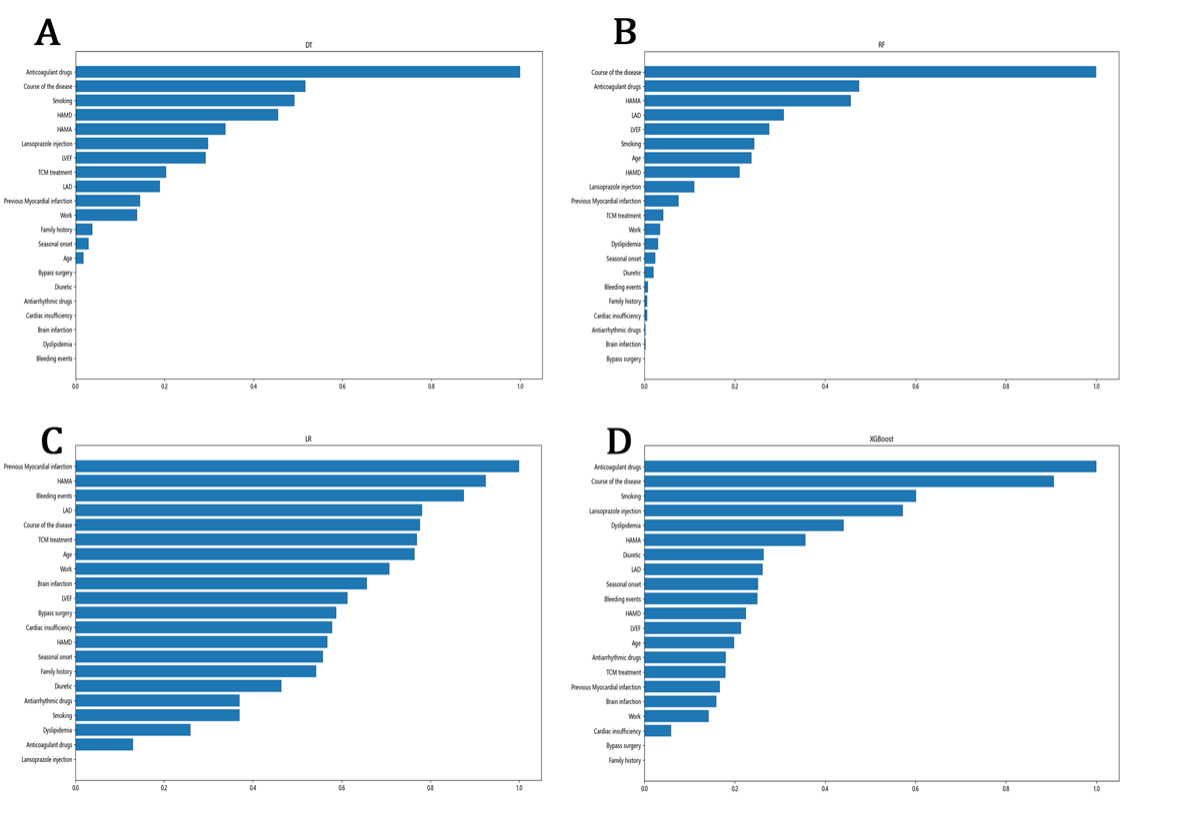
The relative importance of feature variables of the risk prediction models with machine learning algorithms: (A) decision tree (DT), (B) random forest (RF), (C) logistic regression (LR), (D) extreme gradient boosting (XGBoost). TCM: traditional Chinese medicine; LAD: left atrial diameter; LVEF: left ventricular ejection fraction; HAMD: Hamilton depression scale; HAMA: Hamilton anxiety scale.

For a deeper exploration and interpretation of the constructed models, the relative importance of feature variables in each MACE-predicting model is shown in descending order in Figure 3. Since the SVM prediction model used in this study adopted the radial basis function—a complex Gaussian kernel function that makes the SVM model a black box—the direct influence of each feature variable on the SVM model could not be obtained. Similarly, the algorithm of NB used in this study was Gaussian NB. Therefore, only the results of the other ML prediction models are shown.

As shown in Figure 3, the overall trends of DT, RF, and XGBoost demonstrated similar performance, although the relative importance rankings of the three ML models were not completely consistent. More specifically, anticoagulant drugs and the course of disease consistently ranked in the top 2 for all three prediction models. In contrast, the top 2 important features of LR were previous myocardial infarction and HAMA. The relative importance of high-ranking features of XGBoost, the optimal model as a whole, was as follows (in descending order): anticoagulant drugs, course of the disease, smoking, lansoprazole injection, dyslipidemia, HAMA, diuretic, LAD, seasonal onset, bleeding events, HAMD, LVEF, age, antiarrhythmic drugs, TCM treatment, previous myocardial infarction, brain infarction, work, and cardiac insufficiency.

**Table 4:** Comparisons of machine learning algorithms before and after oversampling.

Algorithms		Accuracy	Precision	Recall	F1-score	AUC^a^
**Before oversampling**						
	DT^b^	0.7575	0.5217	0.3200	0.3967	0.7296
	RF^c^	0.7741	0.6667	0.1867	0.2917	0.7888
	LR^d^	0.7608	0.5405	0.2667	0.3571	0.7534
	NB^e^	0.7442	0.4857	0.4533	0.4689	0.7224
	SVM^f^	0.7641	0.7	0.0933	0.1647	0.7431
	XGBoost^g^	0.7807	0.5918	0.3867	0.4677	0.7873
**After oversampling**						
	DT	0.7035	0.73	0.6460	0.6854	0.7748
	RF	0.7522	0.7714	0.7168	0.7431	0.8434
	LR	0.7434	0.7254	0.7832	0.7532	0.7841
	NB	0.7058	0.6598	0.8495	0.7421	0.7463
	SVM	0.7478	0.7593	0.7257	0.7421	0.8075
	XGBoost	0.7788	0.8058	0.7345	0.7685	0.8599

^a^AUC: area under the curve.

^b^DT: decision tree.

^c^RF: random forest.

^d^LR: logistic regression.

^e^NB: naïve Bayes.

^f^SVM: support vector machine.

^g^XGBoost: extreme gradient boosting.

## Discussion

### Principal Results

MACE such as recurrent angina can still occur after coronary revascularization, thus affecting the efficacy and prognosis of surgery. Detecting the postoperative characteristics of patients and combining them with preoperative information to establish a risk assessment model can provide timely warning of the risk of MACE occurrence, thereby helping medical staff and patients to intervene in a timely manner and achieve the purpose of treating the disease before it occurs [37]. In this study, we constructed and evaluated multiple risk models with ML algorithms for MACE prediction in patients within 6 months after coronary revascularization. Performance comparisons of the ML models demonstrated that the XGBoost model performed the best from an overall perspective. Moreover, a deeper exploration of the relative importance of feature variables of the constructed ML models was performed, which is valuable to provide a reference for the pointed intervention and clinical decision-making in MACE prevention.

According to existing studies, the risk factors of MACE after coronary revascularization can be roughly divided into two categories [38]: (1) uncontrollable factors such as gender, age, and family history; and (2) controllable factors such as environment and personal undesirable lifestyle habits. The finding that the risk of MACE occurrence increases with age is similar to that of previous studies [39]. The American Heart Association lists seven major controllable risk factors for coronary heart disease [40]: smoking, physical inactivity, diet, being overweight or obese, abnormal cholesterol levels, high blood pressure, and diabetes. Ritchie et al [41] demonstrated that environment and personal habits contribute to a higher risk of MACE occurrence. Likewise, we found that smokers had a 5.8% higher MACE incidence than that of nonsmokers and mental workers had a 6.9% higher rate than that of manual workers, possibly due to lack of exercise. In addition, we discovered that the occurrence of MACE was correlated with the course of the disease and seasonal changes, which is in line with previous studies [42]. The longer the course of the disease, the higher the incidence of MACE. In addition, the seasonal onset is a reminder of the importance of being proactive in disease prevention according to the seasonal changes in clinical practice.

The secondary prevention of coronary heart disease consists of two main measures: (1) identification and control of risk factors and (2) appropriate drug therapy [43]. For drug therapy, the use of antiplatelet and anticoagulant drugs after coronary revascularization can reduce the incidence of cardiovascular events [44]. The Chinese expert consensus on the clinical application of perioperative nonoral anticoagulants for PCI published in 2018 [45] states that the perioperative period (before, during, and after PCI) is associated with a high incidence of thrombotic events and therefore anticoagulant treatment is important. In this study, the incidence of MACE was lower in patients who were taking anticoagulants than for those without anticoagulants, which was consistent with the findings of Song et al [46] showing that routine anticoagulation treatment after surgery may help to reduce the risk of MACE occurrence. However, anticoagulants can also increase the risk of bleeding [47], especially upper gastrointestinal (UGI) bleeding. It is now generally accepted that proton pump inhibitors have a significant protective effect against UGI bleeding caused by antiplatelet and anticoagulant drugs, with omeprazole and lansoprazole being the most potent inhibitors of CYP2C19 [48], which partly explains the lower incidence of MACE in patients using lansoprazole in our study. Additionally, the univariate analysis showed that the incidence of MACE in patients taking diuretics was 8.9% higher than that of patients who were not taking diuretics, which is consistent with previous research [49,50], and is likely related to the fact that diuretics reduce renal blood flow and increase blood concentrations. Therefore, the use of diuretics should be cautiously considered with a full assessment of the fluid status of patients.

Coronary heart disease belongs to the category of “chest pain” or “heartache” in TCM. The main purpose of treatment is to reduce the incidence of angina pectoris, heart failure, myocardial infarction, and other adverse cardiovascular events [51]. In recent years, clinical practice and related studies have confirmed that TCM treatment has some advantages in relieving angina pectoris, intervening restenosis after PCI, preventing and controlling coronary no-reflow after reperfusion, improving quality of life, increasing exercise tolerance, and reducing the incidence of cardiovascular events and adverse reactions [52,53]. Similarly, we found that the variable of TCM treatment was an important feature in the constructed XGBoost model. Therefore, a combination of TCM and western medicine should be considered to provide more beneficial treatment for MACE prevention in practical clinical decision-making, thereby improving the prevention of MACE after coronary revascularization.

With establishment of the bio-psycho-social medical model, the important role of psychological factors on the occurrence and development of diseases is becoming more widely recognized [54]. A large number of evidence-based medical studies have demonstrated the strong relationship between psychological status and the risk of diseases. Barth et al [55] and Roest et al [56] found that depression and anxiety were important risk factors for morbidity and mortality of patients with coronary heart disease, and Taylor et al [57] suggested that depression, social isolation, and emotional abnormalities were closely associated with the occurrence of cardiovascular disease. Patients with coronary revascularization are more likely to suffer from depression and anxiety due to the dual psychological stress of surgery and underlying diseases, and these adverse psychological responses will directly affect prognosis and eventually become risk factors of MACE. For example, by following up 817 patients undergoing CABG for 5.2 years, Blumenthal et al [58] detected that the mortality of patients with moderate to severe depression was 2 to 3 times higher than that of others within 6 months after surgery. Consistent with these findings, we observed that patients with MACE after coronary revascularization had higher HAMA and HAMD scores, indicating greater levels of anxiety and depression. Consequently, it is important to pay more attention to the mental and psychological state of postoperative patients and provide timely psychological guidance and comfort as needed.

### Limitations

There are several practical deficiencies and limitations of this study. First, the amount of data available for analysis was limited. It is well known that the performance of ML algorithms depends to a certain extent on the sample size and that the model constructed cannot achieve the best performance, and may even be overfitted, with a small data set. In the future, with data supplementation and further research, we will consider more complex ML algorithms, including deep-learning algorithms, to obtain more accurate and efficient prediction models for clinical observation and research. Second, this was a retrospective study from two centers (ie, The People’s Hospital of Liaoning Province and Affiliated Hospital of Liaoning University of Traditional Chinese Medicine). There is a lack of follow-up data on clinical factors and relevant disease progression; thus, a large multicenter sample study is desired for further generalizability and reliability of the results. Last but not least, in addition to numerical structured data such as vital signs and laboratory tests, clinical electronic medical records also contain a massive amount of unstructured data in the form of text such as patients’ complaints, diagnostic records, and medication information; thus, determining the best ways to use such unstructured information for data analysis and modeling will be the focus of future research. Moreover, we plan to integrate structured and unstructured data comprehensively to develop a risk assessment model to predict the risk probability of MACE in patients with coronary heart disease after revascularization.

### Conclusions

In this study, we developed and evaluated risk prediction models for MACE within 6 months after coronary revascularization by utilizing available clinical variables and postoperative follow-up information with ML algorithms. The constructed model can effectively identify high-risk patients with good performance, and the factors that may be associated with MACE were also explored and analyzed in-depth, which is of great significance to provide a reference for medical staff to carry out risk management.

## Appendix

Multimedia Appendix 1 Supplementary Table 1. All variables in univariate analysis.

## Abbreviations

AUC: area under the curve
CABG: coronary artery bypass grafting
DT: decision tree
FN: false negative
FP: false positive
HAMA: Hamilton anxiety scale
HAMD: Hamilton depression scale
IABP: intraaortic balloon pump
LAD: left atrial diameter
LR: logistic regression
LVEF: left ventricular ejection fraction
MACE: major adverse cardiovascular events
ML: machine learning
NB: naïve Bayes
PCI: percutaneous coronary intervention
RF: random forest
ROC: receiver operating characteristic
SMOTE: synthetic minority oversampling technique
SVM: support vector machine
TCM: traditional Chinese medicine
TN: true negative
TP: true positive
UGI: upper gastrointestinal
XGBoost: extreme gradient boosting
