# Supplementary material for: Risk Prediction of Major Adverse Cardiovascular Events Occurrence Within 6 Months After Coronary Revascularization: Machine Learning Study
Source: JMIR Med Inform. 2022 Apr 20;10(4):e33395. doi: 10.2196/33395 (PMC9069286; doi:10.2196/33395)
Supplement: Multimedia Appendix 1 [file medinform_v10i4e33395_app1.docx]

## Multimedia Appendix 1

**Supplementary Table 1.** All variables in univariate analysis.

| Characteristics | | Without MACE^a^ (n=753) | With MACE (n=251) | Statistic^b^ | Degree of freedom | *P* value |
| --- | --- | --- | --- | --- | --- | --- |
| **Gender, n (%)** | | | | .024 | 1 | .9 |
|  | Male | 509 (67.6) | 171 (68.1) |  |  |  |
|  | Female | 244 (32.4) | 80 (31.9) |  |  |  |
| Age (years), mean (SD) | | 63.47 (10.98) | 66.82 (11.10) | -4.180 | 1002 | <.001 |
| BMI^c^, mean (SD) | | 24.79 (3.31) | 24.88 (3.44) | -.376 | 1002 | .7 |
| **Smoking, n (%)** | | | | 4.360 | 1 | .04 |
|  | No | 429 (57.0) | 124 (49.4) |  |  |  |
|  | Yes | 324 (43.0) | 127 (50.6) |  |  |  |
| **Drinking, n (%)** | | | | .016 | 1 | .9 |
|  | No | 561 (74.5) | 188 (74.9) |  |  |  |
|  | Yes | 192 (25.5) | 63 (25.1) |  |  |  |
| **Exercise, n (%)** | | | | .622 | 1 | .4 |
|  | No | 291 (38.6) | 90 (35.9) |  |  |  |
|  | Yes | 462 (61.4) | 161 (64.1) |  |  |  |
| **Type of work, n (%)** | | | | 6.213 | 1 | .01 |
|  | Physical work | 353 (46.9) | 95 (37.8) |  |  |  |
|  | Mental work | 400 (53.1) | 156 (62.2) |  |  |  |
| Course of disease (years since diagnosis), mean (SD) | | 3.41 (5.11) | 5.43 (5.81) | -4.930 | 387.17 | <.001 |
| **Family history, n (%)** | | | | 4.387 | 1 | .04 |
|  | No | 693 (92.0) | 220 (87.6) |  |  |  |
|  | Yes | 60 (8.0) | 31 (12.4) |  |  |  |
| **Seasonal onset, n (%)** | | | | 17.920 | 1 | <.001 |
|  | No obvious seasonality | 675 (89.6) | 199 (79.3) |  |  |  |
|  | Obvious seasonality | 78 (10.4) | 52 (20.7) |  |  |  |
| **Previous myocardial infarction, n (%)** | | | | 80.775 | 1 | <.001 |
|  | No | 643 (85.4) | 147 (58.6) |  |  |  |
|  | Yes | 110 (14.6) | 104 (41.4) |  |  |  |
| **Hypertension, n (%)** | | | | .665 | 1 | .4 |
|  | No | 313 (41.6) | 97 (38.6) |  |  |  |
|  | Yes | 440 (58.4) | 154 (61.4) |  |  |  |
| **Dyslipidemia, n (%)** | | | | 6.659 | 1 | .01 |
|  | No | 681 (90.4) | 240 (95.6) |  |  |  |
|  | Yes | 72 (9.6) | 11 (4.4) |  |  |  |
| **Diabetes, n (%)** | | | | 1.729 | 1 | .2 |
|  | No | 537 (71.3) | 168 (66.9) |  |  |  |
|  | Yes | 216 (28.7) | 83 (33.1) |  |  |  |
| **Brain infarction, n (%)** | | | | 24.822 | 1 | <.001 |
|  | No | 671 (89.1) | 192 (76.5) |  |  |  |
|  | Yes | 82 (10.9) | 59 (23.5) |  |  |  |
| **Arrhythmia, n (%)** | | | | 1.857 | 1 | .2 |
|  | No | 711 (94.4) | 231 (92.0) |  |  |  |
|  | Yes | 42 (5.6) | 20 (8.0) |  |  |  |
| **Cardiac insufficiency, n (%)** | | | | 6.249 | 1 | .01 |
|  | No | 712 (94.6) | 226 (90.0) |  |  |  |
|  | Yes | 41 (5.4) | 25 (10.0) |  |  |  |
| **TCM^d^ treatment, n (%)** | | | | 4.489 | 1 | .03 |
|  | No | 570 (75.7) | 173 (68.9) |  |  |  |
|  | Yes | 183 (24.3) | 78 (31.1) |  |  |  |
| **Nitrate esters, n (%)** | | | | 2.602 | 1 | .1 |
|  | No | 298 (39.6) | 85 (33.9) |  |  |  |
|  | Yes | 455 (60.4) | 166 (66.1) |  |  |  |
| **Beta blockers, n (%)** | | | | .110 | 1 | .7 |
|  | No | 318 (42.2) | 103 (41.0) |  |  |  |
|  | Yes | 435 (57.8) | 148 (59.0) |  |  |  |
| **CCB^e^, n (%)** | | | | 1.294 | 1 | .3 |
|  | No | 550 (73.0) | 174 (69.3) |  |  |  |
|  | Yes | 203 (27.0) | 77 (30.7) |  |  |  |
| **Antiplatelet drugs, n (%)** | | | | .474 | 1 | .5 |
|  | No | 18 (2.4) | 8 (3.2) |  |  |  |
|  | Yes | 735 (97.6) | 243 (96.8) |  |  |  |
| **Anticoagulant drugs, n (%)** | | | | 47.408 | 1 | <.001 |
|  | No | 367 (48.7) | 185 (73.7) |  |  |  |
|  | Yes | 386 (51.3) | 66 (26.3) |  |  |  |
| **Stains, n (%)** | | | | .019 | 1 | .9 |
|  | No | 55 (7.3) | 19 (7.6) |  |  |  |
|  | Yes | 698 (92.7) | 232 (92.4) |  |  |  |
| **ACEI^f^, n (%)** | | | | .708 | 1 | .4 |
|  | No | 644 (85.5) | 220 (87.6) |  |  |  |
|  | Yes | 109 (14.5) | 31 (12.4) |  |  |  |
| **ARB^g^, n (%)** | | | | 3.444 | 1 | .1 |
|  | No | 564 (74.9) | 173 (68.9) |  |  |  |
|  | Yes | 189 (25.1) | 78 (31.1) |  |  |  |
| **Antiarrhythmic drugs, n (%)** | | | | 4.123 | 1 | .04 |
|  | No | 703 (93.4) | 243 (96.8) |  |  |  |
|  | Yes | 50 (6.6) | 8 (3.2) |  |  |  |
| **Diuretic, n (%)** | | | | 6.055 | 1 | .01 |
|  | No | 636 (84.5) | 195 (77.7) |  |  |  |
|  | Yes | 117 (15.5) | 56 (22.3) |  |  |  |
| **Hypoglycemic drugs, n (%)** | | | | .409 | 1 | .5 |
|  | No | 576 (76.5) | 187 (74.5) |  |  |  |
|  | Yes | 177 (23.5) | 64 (25.5) |  |  |  |
| **Sodium phosphocreatine injection, n (%)** | | | | .644 | 1 | .4 |
|  | No | 353 (46.9) | 125 (49.8) |  |  |  |
|  | Yes | 400 (53.1) | 126 (50.2) |  |  |  |
| **Lansoprazole injection, n (%)** | | | | 14.381 | 1 | <.001 |
|  | No | 634 (84.2) | 235 (93.6) |  |  |  |
|  | Yes | 119 (15.8) | 16 (6.4) |  |  |  |
| **Esmolol hydrochloride injection, n (%)** | | | | 2.520 | 1 | .1 |
|  | No | 657 (87.3) | 209 (83.3) |  |  |  |
|  | Yes | 96 (12.7) | 42 (16.7) |  |  |  |
| **Bleeding events, n (%)** | | | | 12.446 | 1 | <.001 |
|  | No | 735 (97.6) | 233 (92.8) |  |  |  |
|  | Yes | 18 (2.4) | 18 (7.2) |  |  |  |
| LAD^h^ (mm), mean (SD) | | 36.59 (4.91) | 37.70 (5.54) | –2.988 | 1002 | .003 |
| RVD^i^ (mm), mean (SD) | | 18.48 (3.52) | 18.22 (1.83) | 1.524 | 827.01 | .1 |
| LVEF^j^ (%), mean (SD) | | 52.65 (8.08) | 51.31 (8.91) | 2.113 | 395.97 | .04 |
| **Stent, n (%)** | | | | 2.616 | 1 | .1 |
|  | No | 55 (7.3) | 11 (4.4) |  |  |  |
|  | Yes | 698 (92.7) | 240 (95.6) |  |  |  |
| **Residual stenosis, n (%)** | | | | 1.159 | 1 | .3 |
|  | No | 636 (84.5) | 219 (87.3) |  |  |  |
|  | Yes | 117 (15.5) | 32 (12.7) |  |  |  |
| **Bypass surgery, n (%)** | | | | 7.200 | 1 | .007 |
|  | No | 745 (98.9) | 242 (96.4) |  |  |  |
|  | Yes | 8 (1.1) | 9 (3.6) |  |  |  |
| HAMD^k^, mean (SD) | | 7.23 (5.26) | 9.27 (5.87) | –4.877 | 392.12 | <.001 |
| HAMA^l^, mean (SD) | | 8.23 (6.59) | 11.13 (6.83) | –5.979 | 1002 | <.001 |

^a^MACE: major adverse cardiovascular events.

^b^*t* statistics for continuous variable comparisons and *χ*^2^ statistics for categorical variables.

^c^BMI: body mass index.

^d^TCM: traditional Chinese medicine.

^e^CCB: calcium channel blockers.

^f^ACEI: angiotensin converting enzyme inhibitor.

^g^ARB: angiotensin receptor blockers.

^h^LAD: left atrial diameter.

^i^RVD: right ventricular diameter.

^j^LVEF: left ventricular ejection fraction.

^k^HAMD: Hamilton depression scale.

^l^HAMA: Hamilton anxiety scale.
